# *Helicobacter pylori* Infection Is Associated with an Increased Risk of Hyperemesis Gravidarum: A Meta-Analysis

**DOI:** 10.1155/2015/278905

**Published:** 2015-03-16

**Authors:** Lin Li, Lingling Li, Xiaoying Zhou, Shuping Xiao, Huiyuan Gu, Guoxin Zhang

**Affiliations:** ^1^Department of Gastroenterology, First Affiliated Hospital of Nanjing Medical University, Nanjing 210029, China; ^2^First Clinical Medical College, Nanjing Medical University, Nanjing 210029, China; ^3^Department of Respiratory Medicine, First Affiliated Hospital of Nanjing Medical University, Nanjing 210029, China

## Abstract

*Background*. Several studies have shown a possible involvement of* Helicobacter pylori *(*H. pylori*) infection in individuals with hyperemesis gravidarum (HG), but the relationship remains controversial. This meta-analysis was performed to validate and strengthen the association between HG and* H. pylori *infection.* Methods*. PubMed, Embase, and Web of Science databases up to March 20, 2014, were searched to select studies on the prevalence of* H. pylori *infection between pregnant women with HG and the normal pregnant control subjects.* Results*. Of the HG cases, 1289 (69.6%) were* H. pylori*-positive; however, 1045 (46.2%) were* H. pylori*-positive in control group. Compared to the non-HG normal pregnant controls, infection rate of* H. pylori *was significantly higher in pregnant women with HG (OR = 3.34, 95% CI: 2.32–4.81, *P* < 0.001). Subgroup analysis indicated that* H. pylori *infection was a risk factor of HG in Asia, Africa, and Oceania, especially in Africa (OR = 12.38, 95% CI: 7.12–21.54, *P* < 0.001).* Conclusions*.* H. pylori *should be considered one of the risk factors of HG, especially in the developing countries.* H. pylori *eradication could be considered to relieve the symptoms of HG in some intractable cases.

## 1. Introduction

Hyperemesis gravidarum (HG), the most severe form of pregnancy-associated nausea and vomiting, is leading to weight loss, nutritional deficiencies, and metabolic disturbance, for example, dehydration, acidosis from starvation, hypokalemia, and transient hepatic dysfunction, often requiring hospitalization and medical treatment to avoid life-threatening complications [1]. HG complicates 0.3–2% of all pregnancies and is a multifactorial disease; however, little is known about the etiology of HG. But a variety of mechanisms may play a role in this disease, such as endocrine factors like human chorionic gonadotropin (HCG), estradiol, and progesterone and immunologic factors, as well as personal factors in which increased body weight has been proposed as possible underlying cause [2]. Recently, several studies have put emphasis on the correlation between* Helicobacter pylori *(*H. pylori*) infection and the risk of HG [3–6].

*H. pylori*, as a gram-negative flagellated spiral bacterium, colonizes stomach and creates the basis of pathogenesis of gastric pathologies, including chronic gastritis, duodenal and gastric ulcers, gastric adenocarcinoma, and mucosa-associated lymphoid tissue lymphoma [7–9].* H. pylori* infected the stomach of 50% of the world population, and it is more prevalent in developing countries [10]. The prevalence of* H. pylori *infection in pregnant women varies according to socioeconomic conditions, geographic area, and even the method used to test* H. pylori* infection.

Several studies have shown a possible involvement of* H. pylori* infection in individuals with HG; however, other studies did not share with them. Although three previous meta-analyses have shown the positive association between* H. pylori* and HG, the role of* H. pylori* infection in the pathogenesis of HG has not reached a consensus. Moreover, the previous meta-analyses did not use the comprehensive search method, unable to include the overall studies, and did not perform a detailed analysis on subgroup to explore the potential factors in HG. On the other hand, the role of some factors such as different populations, geographic areas, ethnicity, and low socioeconomic status is unclear in HG. Therefore, our meta-analysis was undertaken to strengthen the hypothesis that* H. pylori* is a risk factor of HG and to describe the underlying factors in HG.

## 2. Materials and Methods 

### 2.1. Data Sources

We systematically identified studies in PubMed, Embase, and Web of Science (inception through March 20, 2014) databases by two independent investigators (LL and LLL) for all relevant literatures between the risk of HG and the infection of* H. pylori*, by using the MeSH terms, “*Helicobacter pylori*” OR “*H. pylori*” OR “*Helicobacter *infection” AND “hyperemesis gravidarum.” These were searched both as thesaurus terms and as text words. Even “nausea and vomiting” as text word was searched too. Finally, a Google Scholar search was done. Moreover, the references at the end of selected papers were also examined for additional relevant studies.

### 2.2. Inclusion Criteria

In our meta-analysis, the included articles had to meet the following criteria: (1) they must be case-control studies or prospective studies or cross-sectional studies; (2) studies must refer to the association of* H. pylori *infection with HG risk and must provide raw data on* H. pylori* infection in both HG and control groups; (3) the participants must have had a clinical diagnosis of hyperemesis gravidarum as follows: pernicious vomiting, weight loss, and at least one positive ketonuria; (4) the confirmation of* H. pylori* infection was detected by enzyme-linked immunosorbent assay (ELISA), stool antigen test, ^13^C-urea breath test (^13^C-UBT), mucosal biopsy, and polymerase chain reaction (PCR). At least one positive result was considered as confirmation of infection.

### 2.3. Exclusion Criteria

We excluded studies as follows: (1) reports without control groups; (2) reviews and duplicated publications; (3) studies in which the source of* H. pylori* infection in cases and control subjects and other essential information were not offered; hyperthyroidism, multiple gestation trophoblastic neoplasia, gastrointestinal and hepatic disorders, urinary tract or other infections, psychosocial or any other maternal disorders, and any treatment with antacids or antibiotics within the previous 7 days were excluded as well.

### 2.4. Data Extraction

According to the inclusion criteria, data was carefully extracted independently by two reviewers (LL and LLL). For each study analyzed, we collected data including first author, year of publication, country of the population studied, study design, sources of pregnant women (primipara or multipara), gestational age, diagnosis of hyperemesis gravidarum, methods of* H. pylori *detection, total number of persons in cases and controls, and the numbers of* H. pylori-*positive and* H. pylori*-negative patients in the HG group and the control group of each study which were recorded, respectively. For conflicting evaluations, an agreement was reached by consensus and agreement with another reviewer (XYZ), referring back to the original articles.

### 2.5. Statistical Analysis

We used pooled odds ratio (OR) with its corresponding 95% confidence interval (CI) to estimate the strength of the association between HG and* H. pylori *infection. Post hoc subgroup analyses were also performed to explain the heterogeneity in results. Subgroups were explored as follows: detection of* H. pylori *infection (serum* H. pylori* IgG/IgM/IgA antibody by ELISA, stool antigen test, mucosal biopsy from endoscopy, or* H. pylori* genome by PCR), publication period (1996–2000, 2001–2005, 2006–2010, or 2011–2014), and region (Asia, North America, Europe, Africa, or Oceania).

The heterogeneity of the studies included in this meta-analysis was assessed using the *Q* statistic test and the *I* ^2^ statistic test. The random-effects model was selected when *P* value < 0.1 or *I* ^2^ > 50%; otherwise, the fixed-effects model was selected. Possible publication bias was evaluated by visual inspection of funnel plots and application of Begg's and Egger's test [11–15]. *P* values of less than 0.05 from Egger's test were considered statistically significant.

All statistical analyses were done with STATA statistical software package version 12.0 (2000; STATA Corp., College Station, TX, USA); *P* < 0.05 was identified as statistically significant.

## 3. Results

### 3.1. Literature Search

**Figure 1:**
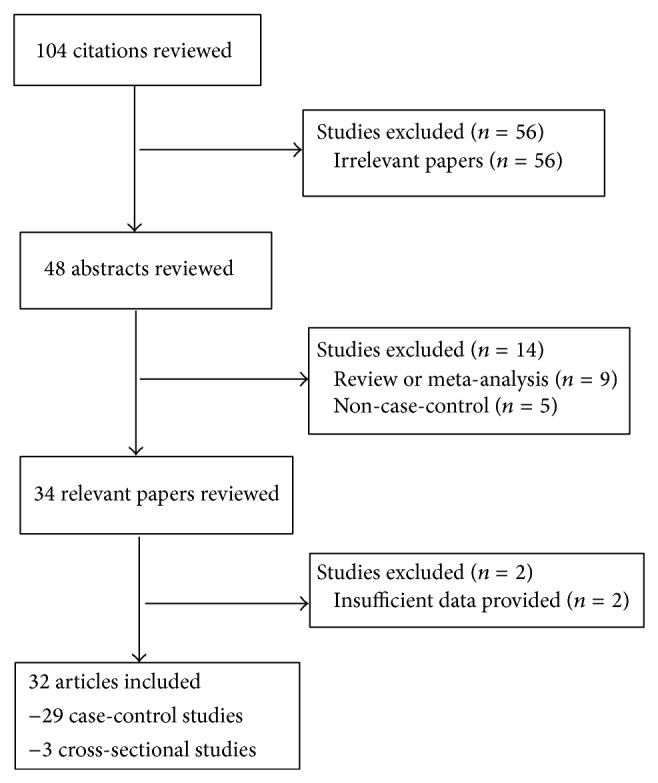
Flow chart of the literature searches for evaluating* Helicobacter pylori* infection and hyperemesis gravidarum.

As shown in Figure 1, after rigorous searching, we identified 104 citations. Of these, fifty-six irrelevant papers were excluded after screening the titles. In the remaining 48 articles, 16 studies included five studies without control group, two did not provide sufficient data, and 9 reviews or meta-analyses were discarded. Thus, a total of thirty-two studies included twenty-nine case-control studies and three cross-sectional studies published between 1998 and 2014 fulfilled our inclusion criteria and were included in the meta-analysis [3–6, 16–43].

### 3.2. Characteristics of Included Studies

With respect to the* H. pylori* detection methods, serum* H. pylori* IgG/IgM/IgA antibody was detected by ELISA in twenty-nine, one, and one studies, respectively. However,* H. pylori* stool antigen (HpSA) was used in seven articles,* H. pylori* genome by PCR (Hp PCR DNA) tested in two studies, and biopsy and histological examination from endoscopy in one literature. Taking into account publication period, five studies were published from 1996 to 2000, twelve researches were from 2001 to 2005, eight studies were from 2006 to 2010, and seven articles were from 2011 to 2014. In addition, in terms of region, twenty studies were from Asian countries (Turkey, Iran, Japan, and China were grouped in Asia according to similarities in geographic position and racial traits), five from North America (USA, Canada, and Puerto Rico were grouped in North America according to similarities in geographic location and racial traits), three from Europe (Norway and Greece were grouped in Europe according to similarities in geographic position and racial traits), three from Africa, and one from Oceania. Apparently, all studies used hospital-based controls. All of the studies followed a standard definition of HG excluding subjects with differential diagnosis such as infections, gastrointestinal and endocrine diseases, or psychiatric illness.

### 3.3. Overall

**Figure 2:**
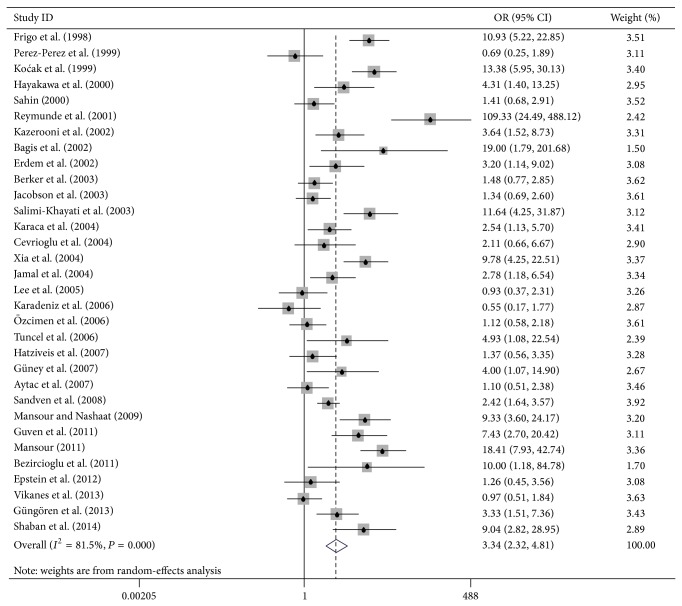
Meta-analysis for the association between HG and* Helicobacter pylori* infection.

Thirty-two articles published between 1998 and 2014 met the inclusion criteria and their characteristics were shown in Tables 1 and 2 in detail. In total, thirteen studies found no association between* H. pylori* infection and the risk of HG [12, 18, 20, 26, 27, 32–34, 36, 38, 41–43]. However, nineteen researches suggested* H. pylori* infection during pregnancy might be a risk factor for pregnant women with HG [3–6, 16, 17, 19, 21, 23–25, 28–31, 35, 37, 39, 40]. All included studies contained a total sample size of 4113 patients and contained 1851 HG cases and 2262 controls, with a total* H. pylori* infection rate of 56.7% (2334/4113). Of the HG cases, 1289 (69.6%) were* H. pylori*-positive; however, 1045 (46.2%) were* H. pylori*-positive in control group. Since overall *I* ^2^ was 81.5%, we used the random-effects model for our analysis. As shown in Figure 2, the overall OR was 3.34 (95% CI: 2.32–4.81) and the overall effect *Z* value was 6.51 (*P* < 0.001), which indicated that there was a powerful association between* H. pylori* infection and risk of HG.

### 3.4. Subgroup Analysis

To evaluate the underlying confounding factors that may have impacted the overall results, we further conducted subgroup analyses based on detection of* H. pylori* infection, publication period, and region, respectively. Since *I* ^2^ were 0 in subgroups of Hp PCR DNA test and Africa, we selected the fixed-effects model. In the remaining subgroups, *I* ^2^ were >50%, so the random-effects models were used. As shown in Table 3, ORs of serum* H. pylori* IgM/IgA antibody test by ELISA were 7.77 (95% CI: 0.43–140.84) and 1.32 (95% CI: 0.56–3.12), respectively, which suggested that there was no association between* H. pylori* and HG. However, serum* H. pylori* IgG antibody test by ELISA (OR = 3.32, 95% CI: 2.28–4.84), HpSA test (OR = 1.88, 95% CI: 1.33–2.65), and Hp PCR DNA test (OR = 5.87, 95% CI: 2.47–13.93) reflected* H. pylori* infection was a risk factor of HG. In addition, method of histological examination from endoscopy also showed a positive correlation between* H. pylori* infection and HG (OR = 19.0, 95% CI: 1.79–201.68). With respect to publication period, studies from 1996 to 2000 (OR = 3.66, 95% CI: 1.17–11.48), 2001 to 2005 (OR = 3.89, 95% CI: 2.10–7.21), 2006 to 2010 (OR = 2.01, 95% CI: 1.15–3.49), and 2011 to 2014 (OR = 4.41, 95% CI: 1.72–11.29) all showed significantly high rates of* H. pylori* infection in pregnant women with HG compared to those with normal pregnancy (Table 3). In the subgroup of region, shown in Table 3, compared to those studies in North America (OR = 2.33, 95% CI: 0.63–8.62) and Europe (OR = 1.55, 95% CI: 0.83–2.91), researches of Asia (OR = 3.27, 95% CI: 2.18–4.91), Africa (OR = 12.38, 95% CI: 7.12–21.54), and Oceania (OR = 10.93, 95% CI: 5.22–22.85) reflected* H. pylori* was positively related to HG.

### 3.5. Bias Diagnostics

**Figure 3:**
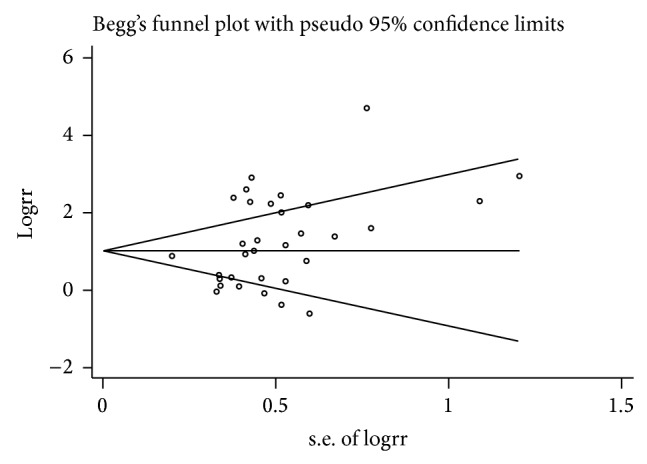
Publication bias tests for the overall data (*H. pylori-*positive versus* H. pylori-*negative).

Begg's test was created for assessment of possible publication bias (Figure 3). The *P* values for Egger's tests were *P* = 0.067 (*P* > 0.05), indicating the absence of heterogeneity and implying that the results of the present meta-analysis were relatively stable and that the publication bias might exert little influence on the overall results.

## 4. Discussion

### 4.1. Main Findings

This updated meta-analysis suggests that exposure to* H. pylori* is associated with an increased risk of HG. The studies included in this meta-analysis, containing 1851 HG patients of which 1289 cases were confirmed with* H. pylori* infection, implied that the rate of* H. pylori* infection was much greater in HG patients (1289/1851) than that in non-HG patients (1045/2262) after adjusting for confounding variables (*P* < 0.001).

Our meta-analysis contained 32 articles including 29 case-control studies and 3 cross-sectional articles, which contained comprehensive articles and added new primary studies. We enrolled much more patients with HG (1851) and controls (2262) than those in published studies to further confirm this relationship. We separated subgroups in detection of* H. pylori *infection, publication period, and region to comprehensively evaluate the underlying confounding factors that may have impacted the overall results. As reported before, three previous articles reported similar meta-analysis results [44–46]. Golberg et al. [44] performed a meta-analysis of 14 studies and did subgroup analysis only on markers of* H. pylori* infection, which suggested that the association might be possible if testing methods for active* H. pylori* infection used nonserological methods. However, our meta-analysis showed that the serological method such as* H. pylori* IgG antibody test was relatively credible. Sandven et al. [45] included 25 case-control studies containing 1455 HG and 1970 controls and carried out subgroups analyses on matched design with nonmatched design and Turkish population with other population. We separated subgroup analyses of region according to Asia, North America, Europe, Africa, or Oceania as described above. Niemeijer et al. [46] performed a systematic review to summarize evidence on biomarkers of HG and their value in diagnosis and estimating disease severity and carried out a diagnostic meta-analysis of 19 studies on* H. pylori* IgG, which showed a sensitivity of 73% and a specificity of 55% in the diagnosis of HG as compared to controls without HG. Our study paid attention not only to the detection method of* H. pylori* IgG, but also to the methods of Hp PCR DNA and HpSA. We also described other detection methods used in pregnant women.

With respect to detection methods used in pregnant women, Hp PCR DNA test seemed much more efficient than ELISA test of serum* H. pylori* IgG antibody, and the latter was more reliable than HpSA test. Of note, Cevrioglu et al. [30] collected both serum and feces samples from pregnant women with HG to investigate specific antibodies for* H. pylori *(immunoglobulin-IgG, IgA) and HpSA, and only HpSA test suggested significant association between* H. pylori* infection and HG while serologic assessment failed to reflect the association (40% versus 12.4%, *P* < 0.001). By using PCR with specimen of saliva, Güngören et al. [42] found a positive relationship between the symptoms of HG and* H. pylori* positivity, while test of* H. pylori* IgG/IgM antibody failed to detect this association between the symptoms of HG and* H. pylori* positivity. From biopsies of the gastric antrum and corpus, Bagis et al. [23] found that, compared to controls, HG patients were detected with higher* H. pylori* density, degree of inflammation, and* H. pylori* activation, implying that* H. pylori* density might be related to HG since the bacterium density of controls was lower. These results suggested that the degree of gastric complaints might be related to density of* H. pylori*.

In addition, two studies from New Zealand indicate that incidence of hyperemesis gravidarum differs according to women's ethnic origin [47, 48]. Just as shown in our subgroup analysis of region, studies in North America and Europe suggested no association between* H. pylori* infection and risk of HG, while in Asia, Africa, and Oceania analysis indicated* H. pylori* infection was a risk factor of HG, especially in Africa. Sandven et al. [39] also found this association between* H. pylori* infection and HG was much stronger in Africans as compared to non-Africans. This might be explained by ethnicity and low socioeconomic status.* H. pylori*-infected Africans possibly carry an aggressive variant of the bacterium and the host immune mechanisms might be a key to different responses to* H. pylori* in different populations and geographic areas. As well documented, the prevalence rate of* H. pylori* infection is much higher in developing countries than that in developed countries [49–51]. As Eshraghian reviewed, the overall prevalence of* H. pylori* infection in Iran and other Eastern Mediterranean Regional Office countries such as Egypt and Afghanistan, irrespective of time and age group, ranged from 30.6% to 82% and ranged from 22% to 87.6%, respectively. However the* H. pylori *prevalence in North Africa was 76% [52]. The prevalence is high in developing countries, while pregnant women with HG in these countries have higher rate of* H. pylori *infection. For example, it is 50%–70% in Turkey [53], more than 80% in Egypt [54]. The above studies all suggested that* H. pylori *infection was a risk factor of HG. Strategies to improve sanitary facilities, educational status, and socioeconomic status should be implemented to minimize* H. pylori* infection and come into the result of decline prevalence of HG.

Our meta-analysis suggested that* H. pylori *infection was a risk factor of HG. Frigo et al. suggested that the* H. pylori *may contribute to its persistence beyond the normal time course [16]. It was once reported that HG was an oxidative stress condition induced by increasing reactive oxygen species (ROS) activity and decreasing antioxidant status [55]. Meanwhile,* H. pylori* colonizes gastric mucosa and generates ROS as well as downregulating levels of plasma antioxidants such as ascorbic acid [7], similar to Güney et al. [37] who found that, compared to the control, level of serum malondialdehyde (MDA) was significantly higher and activities of antioxidant enzymes such as superoxide dismutase (SOD), catalase (CT), and glutathione peroxidase (GSH-Px) were significantly lower in the HG group (*P* < 0.01). Therefore, they hypothesized that increased ROS activity or decreased antioxidant potential, possibly induced by* H. pylori*, might have a pathogenic role in HG. To date, however, the knowledge of how* H. pylori* causes HG is still very limited, and we assumed the following. Firstly, hormonal mechanisms, in the early phase of pregnancy, as a result of the elevated steroid and human chorionic gonadotropin (HCG) levels, accumulation of fluid, and a displacement of intracellular and extracellular volume occur which in turn leads to a shift in pH in the gastrointestinal tract during pregnancy [40, 56]. Secondly, emotional factors, the moods of pregnant women change frequently due to the changes of endocrine hormones that might increase women's susceptibility to infection caused by altered cell-mediated immunity that causes changes of various classes of antibodies during different gestational periods [57, 58]. Thirdly,* H. pylori* infection might be one potential reason for HG. Dysmotility of gastrointestinal tract and prolonged gastric emptying and intestinal transit time induced by pregnancy might favor infectionof* H. pylori* [56, 58]. On the other side, host inflammation response to varies of virulence of* H. pylori *strains also different from each other. The virulence of the organism might be another factor creating a possible link between* H. pylori *and the precipitation of HG. As we all know, cytotoxin-associated gene A product (CagA) and vacuolating cytotoxin A (VacA) are used as markers for genomic diversity of* H. pylori*. In Western countries VacA, rather than CagA, was associated with more severe diseases, while in East Asian countries it is the opposite [59–61]. Similarly, as shown in Table 4, Xia et al. in Asia (OR = 13.96, 95% CI: 6.56–29.71) found that prevalence of* H. pylori *infection with CagA was positively correlated with HG [29]. Nevertheless Perez-Perez et al. in USA [18] failed to find that CagA plays a role in HG. To date, Vikanes et al. [43] conducted the first case-control study to examine the relationship between* H. pylori* and HG by both CagA and VacA seropositivity in Norway and their results suggested that CagA and VacA seropositivity were not significantly associated with HG. So the role of CagA and VacA in the pathopoiesis of HG was still unclear, and no more studies were performed to explore this relationship. It suggested that study of CagA and VacA might be generalized and probably aim at the populations with high prevalence rate of* H. pylori *infection.

On the other hand, eradication of* H. pylori* could relieve the symptoms of HG. Strachan et al. reported a case of eradication in a 38-year-old woman in her third pregnancy, who orally omeprazole 20 mg bid, metronidazole 400 mg bid, and amoxicillin 500 mg tid for 7 days in her 30 weeks of pregnancy. This led to prompt resolution of her vomiting and improvement of her reflux symptoms [62]. El Younis et al. also reported two cases in which first-trimester patients with severe HG required intravenous fluid replacement. Rapid improvement of the HG was observed with complete resolution of all symptomatology after using of erythromycin therapy orally, which possibly suggests a new therapeutic modality for similar patients [63]. On the other hand, omeprazole is not licensed for use in pregnancy and ought to be used with caution although experience from case-control and observational studies has not revealed any increase in congenital malformations or pregnancy complications [64]. Large-scale studies were not performed. However, the above case reports could verify that the eradication of* H. pylori* might relieve the symptoms of HG.

### 4.2. Interpretation

This meta-analysis has some limitations to be acknowledged. Firstly, our meta-analysis only focused on papers published in the English language and might miss some eligible studies that were unpublished in other languages. Secondly, the articles identified were limited to those openly published up to March 2014, and it is possible that some related published or unpublished studies that might meet the inclusion criteria were missed. Finally, despite using a precise literature searching strategy to identify eligible studies, it is possible that a few studies meeting the inclusion criteria were not included, resulting in any inevitable bias, though the funnel plots and Egger's tests failed to show any significant publication bias.

## 5. Conclusion

In conclusion, our meta-analysis suggested that there was a strong association between* H. pylori* infection and HG, allowing us to conclude that* H. pylori *should, therefore, be considered as one of the risk factors of HG. Screening for* H. pylori* should be added to the investigations for HG, especially in the developing countries. Appropriate therapeutic regimens for eradication of* H. pylori* could be considered to relieve the symptoms of HG in some intractable cases.

## Figures and Tables

**Table 1:** Characteristics of studies on *H. pylori* infection in HG cases and controls.

First author, year	Study design	Country	Sample size	HG definition	Detection method of *H. pylori *	Gestational age when included in the study	Sources of pregnant women
Frigo, 1998 [16]	Case-control	Austria	234	Vomiting >3 times/day weight loss >3 kg ketonuria	Serum IgG antibodies by FEI	—	—

Perez-Perez, 1999 [18]	Case-control	USA	89	Vomiting >3 times/day weight loss >3 kg ketonuria	Serum cagA by ELISA	—	—

Koćak, 1999 [17]	Case-control	Turkey	211	Vomiting >3 times/day ketonuria	Serum IgG antibodies by FEI	7–16 weeks	141 Primigravida 70 Multigravida

Hayakawa, 2000 [19]	Case-control	Japan	63	Nausea and vomiting in first trimester	Serum IgG antibodies by ELISA	The first trimester	—

Sahin, 2000 [20]	Case-control	Turkey	120	Prolonged severe vomiting dehydration weight loss >5% ketonuria	Serum IgG antibodies by ELISA	—	—

Reymunde, 2001 [21]	Case-control	Puerto Rico	89	Daily nausea and vomiting in gestation weeks 6–16	Serum IgG antibodies not specified	6–16 weeks	—

Kazerooni, 2002 [24]	Case-control	Iran	107	Vomiting >3 times/day weight loss >3 kg ketonuria	Serum IgG antibodies by ELISA	7–16 weeks	62 Primigravida 45 Multigravida

Bagis, 2002 [23]	Case-control	Turkey	30	Severe HG in the first 16 weeks of pregnancy	Endoscopy with biopsy + histological examination	16 weeks	—

Erdem, 2002 [22]	Case-control	Turkey	86	Vomiting >4 times/day weight loss >5% ketonuria	Serum IgG antibodies by ELISA	—	—

Berker, 2003 [27]	Case-control	Turkey	240	Vomiting >3 times/day weight loss >5% ketonuria	Serum IgG antibodies by ELISA	10–14 weeks	55 Primigravida 105 Multigravida

Jacobson, 2003 [26]	Case-control	Canada	206	Vomiting >3 times/day weight loss ketonuria	Serum IgG antibodies by ELISA	7–19 weeks	—

Salimi-Khayati, 2003 [25]	Case-control	Iran	108	Vomiting >3 times/day weight loss >5% ketonuria	Serum IgG antibodies by ELISA	6–16 weeks	93 Primigravida 15 Multigravida

Karaca, 2004 [31]	Case-control	Turkey	146	Vomiting >3 times/day weight loss >5% ketonuria	Serum IgG antibodies by FEI	—	—

Cevrioglu, 2004 [30]	Case-control	Turkey	124	Vomiting >3 times/day weight loss >5% ketonuria	Serum IgG antibodies by ELISA	The first 16 weeks	—

Xia, 2004 [29]	Case-control	China	172	Vomiting >3 times/day weight loss >3 kg ketonuria	Serum specific IgG antibodies and CagA by ELISA	7–16 weeks	172 Multigravida

Jamal, 2004 [28]	Case-control	Iran	94	Vomiting >3 times/day weight loss >3 kg ketonuria	Serum IgG antibodies by ELISA	7–16 weeks	49 Primigravida 45 Multigravida

Lee, 2005 [32]	Cross-sectional	USA	82	Vomiting >3 times/day weight loss >5% ketonuria	Serum IgG antibodies by ELISA	—	—

Karadeniz, 2006 [34]	Case-control	Turkey	60	Vomiting >3 times/day weight loss >5% ketonuria	Serum IgG antibodies by ELISA	8–14 weeks	—

Özcimen, 2006 [33]	Cross-sectional	Turkey	140	Vomiting >3 times/day weight loss >3 kg ketonuria	Serum IgG antibodies by ELISA	—	—

Tuncel, 2006 [35]	Case-control	Turkey	138	Vomiting >3 times/day weight loss >3 kg ketonuria	Serum IgG antibodies by ELISA	—	—

Hatziveis, 2007 [38]	Case-control	Greece	110	Vomiting >3 times/day weight loss >3 kg ketonuria	Serum IgG antibodies by ELISA	8–14 weeks	39 Primigravida 71 Multigravida

Güney, 2007 [37]	Case-control	Turkey	60	Vomiting >4 times/day weight loss >5% ketonuria	Serum IgG antibodies not specified	7–12 weeks	—

Aytac, 2007 [36]	Case-control	Turkey	107	Vomiting >3 times/day weight loss >5% ketonuria	Stool antigen by ELISA	8–13 weeks	—

Sandven, 2008 [39]	Case-control	Norway	488	ICD-9 Code 643.1	Serum IgG antibodies by EIA		

Mansour, 2009 [40]	Case-control	Egypt	100	Vomiting >3 times/day weight loss ketonuria	Serum IgG antibodies by ELISA	10–16 weeks	67 Primigravida 33 Multigravida

Guven, 2011 [5]	Cross-sectional	Turkey	80	Vomiting >3 times/day weight loss >5% ketonuria dehydration	Serum IgG antibodies by ELISA	7–12 weeks	—

Mansour, 2011 [4]	Case-control	Egypt	160	Vomiting >3 times/day weight loss >3 kg ketonuria	Serum IgG antibodies by ELISA	10–16 weeks	—

Bezircioğlu, 2011 [3]	Case-control	Turkey	72	Vomiting >4 times/day weight loss ≥3 kg ketonuria	Stool antigen by ELISA	10–14 weeks	—

Epstein, 2012 [41]	Case-control	USA	82	Met the diagnosis of hyperemesis	Serum IgG antibodies by ELISA	—	—

Vikanes, 2013 [43]	Case-control	Norway	170	Long-lasting nausea and vomiting, weight loss, electrolyte disturbances	Serum IgG antibodies and CagA and VacA by EIA	17th and 22nd	—

Güngören, 2013 [42]	Case-control	Turkey	140	Vomiting 3 times/day weight loss ketone	Serum IgG antibodies by ELISA	Below the 20th week	—

Shaban, 2014 [6]	Case-control	Egypt	100	Vomiting >3 times/day weight loss >5% or 3 kg ketonuria	Serum IgG antibodies by membrane-based immunoassay	5–12 weeks	36 Primigravida 64 Multigravida

HP: *Helicobacter pylori*; HG: hyperemesis gravidarum; FEI: fluorescent enzyme immunoassay; EIA: enzyme immunoassay; ELISA: enzyme-linked immunosorbent assay.

**Table 2:** Statistical analysis of studies on the rate of *H. pylori* infection in HG cases and non-HG controls.

First author, year	Number of cases	Number of controls	Number of cases with HP (+), *n* (%)	Number of controls with HP (+), *n* (%)	OR (95% CI)
Frigo, 1998 [16]	105	129	95 (90.5)	60 (46.5)	10.93 (5.22, 22.85)
Perez-Perez, 1999 [18]	42	47	8 (19.1)	12 (25.5)	0.69 (0.25, 1.89)
Koćak, 1999 [17]	95	116	87 (91.5)	52 (44.8)	13.38 (5.95, 30.13)
Hayakawa, 2000 [19]	34	29	18 (52.9)	6 (20.6)	4.31 (1.40, 13.25)
Sahin, 2000 [20]	60	60	37 (61.7)	32 (53.3)	1.41 (0.68, 2.91)
Reymunde, 2001 [21]	45	44	40 (89.0)	3 (7.0)	109.33 (24.49, 488.12)
Kazerooni, 2002 [24]	54	53	44 (81.5)	29 (54.7)	3.64 (1.52, 8.73)
Bagis, 2002 [23]	20	10	19 (95.0)	5 (50.0)	19.00 (1.79, 201.68)
Erdem, 2002 [22]	47	39	40 (85.1)	25 (64.1)	3.20 (1.14, 9.02)
Berker, 2003 [27]	80	80	56 (70.0)	49 (61.2)	1.48 (0.77, 2.85)
Jacobson, 2003 [26]	53	153	19 (35.7)	45 (29.7)	1.34 (0.69, 2.60)
Salimi-Khayati, 2003 [25]	54	54	48 (88.9)	22 (40.7)	11.64 (4.25, 31.87)
Karaca, 2004 [31]	56	90	46 (82.1)	58 (64.4)	2.54 (1.13, 5.70)
Cevrioglu, 2004 [30]	27	97	23 (85.2)	71 (73.2)	2.11 (0.66, 6.67)
Xia, 2004 [29]	72	100	64 (88.9)	45 (45.0)	9.78 (4.25, 22.51)
Jamal, 2004 [28]	39	55	26 (66.7)	23 (41.8)	2.78 (1.18, 6.54)
Lee, 2005 [32]	40	42	26 (65.0)	28 (66.7)	0.93 (0.37, 2.31)
Karadeniz, 2006 [34]	31	29	21 (67.7)	23 (79.3)	0.55 (0.17, 1.77)
Özcimen, 2006 [33]	70	70	35 (50.0)	33 (47.1)	1.12 (0.58, 2.18)
Tuncel, 2006 [35]	50	88	48 (96.0)	73 (82.9)	4.93 (1.08, 22.54)
Hatziveis, 2007 [38]	25	85	14 (56.0)	41 (48.2)	1.37 (0.56, 3.35)
Güney, 2007 [37]	25	20	20 (80.0)	10 (50.0)	4.00 (1.07, 14.90)
Aytac, 2007 [36]	52	55	22 (42.3)	22 (40.0)	1.10 (0.51, 2.38)
Sandven, 2008 [39]	244	244	105 (43.0)	58 (23.8)	2.42 (1.64, 3.57)
Mansour, 2009 [40]	50	50	42 (84.0)	18 (36.0)	9.33 (3.60, 24.17)
Guven, 2011 [5]	40	40	32 (80.0)	14 (35.0)	7.43 (2.70, 20.42)
Mansour, 2011 [4]	80	80	71 (88.8)	24 (30.0)	18.41 (7.93, 42.74)
Bezircioğlu, 2011 [3]	36	36	8 (22.2)	1 (2.8)	10.00 (1.18, 84.78)
Epstein, 2012 [41]	23	59	16 (69.6)	38 (64.4)	1.26 (0.45, 3.56)
Vikanes, 2013 [43]	62	108	38 (61.3)	67 (62.0)	0.97 (0.51, 1.84)
Güngören, 2013 [42]	90	50	75 (83.3)	30 (60.0)	3.33 (1.51, 7.36)
Shaban, 2014 [6]	50	50	46 (92.0)	28 (56.0)	9.04 (2.82, 28.95)

HG: hyperemesis gravidarum; OR: odds ratio; CI: confidence interval.

**Table 3:** Subgroup analysis of the prevalence of *H. pylori *infection in HG cases versus controls.

Subgroup	Number of studies	Cases with HP (+)	Controls with HP (+)	OR [95% CI]	*P*	*Z*	Tests of heterogeneity			
							*Q*	df	*P*	*I* ^2^ (%)
Region										
Asia	20	769/1017	593/1121	3.27 [2.18, 4.91]	<0.001	5.71	68.04	19	<0.001	72.1
North America	5	109/203	28/345	2.33 [0.63, 8.62]	0.205	1.27	36.55	4	<0.001	89.1
Europe	3	157/331	166/437	1.55 [0.83, 2.91]	0.170	1.37	6.18	2	0.046	67.6
Africa	3	159/180	70/180	12.38 [7.12, 21.54]	<0.001	8.91	1.47	2	0.479	0
Oceania	1	95/105	60/129	3.34 [2.32, 4.81]	<0.001	6.35	0.00	0	—	—
Year										
1996–2000	5	245/336	162/381	3.66 [1.17, 11.48]	0.026	2.23	35.41	4	<0.001	88.7
2001–2005	12	451/587	403/817	3.89 [2.10, 7.21]	<0.001	4.32	57.01	11	<0.001	80.7
2006–2010	8	307/547	278/641	2.01 [1.15, 3.49]	0.014	2.47	24.07	7	0.001	70.9
2011–2014	7	286/381	202/423	4.41 [1.72, 11.29]	0.002	3.09	39.71	6	<0.001	84.9
HP testing method										
HpIgGAb	29	1240/1743	1017/2161	3.32 [2.28, 4.84]	<0.001	6.25	158.05	28	<0.001	82.3
HpSA	7	124/300	124/435	1.88 [1.33, 2.65]	0.0004	3.57	9.50	6	0.091	47.4
HpDNA PCR	2	45/124	10/79	5.87 [2.47, 13.93]	<0.001	4.02	0.63	1	0.43	0
HpIgAAb	1	13/27	40/97	1.32 [0.56, 3.12]	0.52	0.64	0.00	0	—	—
HpIgMAb	1	6/90	0/50	7.77 [0.43, 140.84]	0.17	1.39	0.00	0	—	—
histologically	1	19/20	5/10	19.00 [1.79, 201.68]	0.01	2.44	0.00	0	—	—
All studies	32	1289/1851	1045/1217	3.34 [2.32, 4.81]	<0.001	6.51	167.81	31	<0.001	81.5

HP:* Helicobacter pylori*; HG: hyperemesis gravidarum; OR: odds ratio; CI: confidence interval; df: degrees of freedom; HpIgGAb: *H. pylori* IgG antibodies; HpIgAAb: *H. pylori* IgA antibodies; HpIgMAb: *H. pylori* IgM antibodies; HpSA: *H. pylori *stool antigen; Hp DNA PCR: *H. pylori* genome by PCR.

**Table 4:** The infection rate of CagA- or VacA-positive *H. pylori* in HG cases and controls.

First author, year	Region	Number of cases	Number of controls	Number of cases with CagA- and/or VacA-positive *H. pylori*, *n* (%)	Number of controls with CagA- and/or VacA-positive *H. pylori*, *n* (%)	OR (95% CI)
Xia, 2004 [29]	Asia	72	100	50 (69.4%)	14 (14%)	13.96 (6.56, 29.71)
Vikanes, 2013 [43]	Europe	62	108	21 (33.9%)	44 (40.8)	0.75 (0.39, 1.43)

CagA: cytotoxin-associated gene A product; VacA: vacuolating cytotoxin A; HG: hyperemesis gravidarum; OR: odds ratio; CI: confidence interval.
